# Association Mapping in Scandinavian Winter Wheat for Yield, Plant Height, and Traits Important for Second-Generation Bioethanol Production

**DOI:** 10.3389/fpls.2015.01046

**Published:** 2015-11-26

**Authors:** Andrea Bellucci, Anna Maria Torp, Sander Bruun, Jakob Magid, Sven B. Andersen, Søren K. Rasmussen

**Affiliations:** Plant and Soil Section, Department of Plant and Environmental Sciences, Faculty of Science, University of CopenhagenFrederiksberg, Denmark

**Keywords:** QTL, *Triticum aestivum* L., GWAS, recalcitrance, ligno-cellulosic biomass

## Abstract

A collection of 100 wheat varieties representing more than 100 years of wheat-breeding history in Scandinavia was established in order to identify marker-trait associations for plant height (PH), grain yield (GY), and biomass potential for bioethanol production. The field-grown material showed variations in PH from 54 to 122 cm and in GY from 2 to 6.61 t ha^-1^. The release of monomeric sugars was determined by high-throughput enzymatic treatment of ligno-cellulosic material and varied between 0.169 and 0.312 g/g dm for glucose (GLU) and 0.146 and 0.283 g/g dm for xylose (XYL). As expected, PH and GY showed to be highly influenced by genetic factors with repeatability (*R*) equal to 0.75 and 0.53, respectively, while this was reduced for GLU and XYL (*R* = 0.09 for both). The study of trait correlations showed how old, low-yielding, tall varieties released higher amounts of monomeric sugars after straw enzymatic hydrolysis, showing reduced recalcitrance to bioconversion compared to modern varieties. Ninety-three lines from the collection were genotyped with the DArTseq^®^ genotypic platform and 5525 markers were used for genome-wide association mapping. Six quantitative trait loci (QTLs) for GY, PH, and GLU released from straw were mapped. One QTL for PH was previously reported, while the remaining QTLs constituted new genomic regions linked to trait variation. This paper is one of the first studies in wheat to identify QTLs that are important for bioethanol production based on a genome-wide association approach.

## Introduction

Second-generation bioethanol production based on ligno-cellulosic material from agricultural waste and plant residues is not undertaken specifically for the purposes of bioenergy, and therefore has the potential to bypass the food–feed–fuel dilemma which is an inherent problem with other kinds of bioenergy (Oladosu and Msangi, 2013). With an estimated annual production in Europe of 74 million tons (Kretschmer et al., 2012), wheat (*Triticum aestivum L.*) straw is the crop residue with the greatest potential to be a feedstock for second-generation biofuels available in Europe (Scarlat et al., 2010). If the straw is used for bioenergy, wheat can be regarded as a dual-purpose crop, providing grains for food and feed and remaining biomass for energy. Then, it would be desirable to increase straw production without compromising grain yield (GY). However, it is also advantageous to have straw that is easy to convert, as this is more efficient and requires fewer inputs for transformation. Therefore, another target would be to develop wheat varieties with straw that is less recalcitrant for bioconversion. This could require alterations in the lignin and secondary cell wall composition. The biosynthesis of monolignols is well known and several examples of genetic modification by targeting single genes have been pursued in model and crop plant species (Sibout et al., 2005; Chen and Dixon, 2007; Fu et al., 2011), but not for wheat as a crop. For secondary cell wall biosynthesis, knowledge is more fragmented and crop specific details are scarce or missing. A meta-analysis of 150 quantitative trait loci (QTLs) for cell wall composition identified in 11 maize mapping populations resulted in 42 mQTL, showing many genes influencing the cell wall that are stable across varieties and environments (Truntzler et al., 2010).

Plant height (PH) is one of the most studied phenotypes in wheat due to its involvement in plant architecture and ultimately in GY. In fact, height is also closely related to recalcitrance, because shorter straw tends to have a more easily degradable leaf mass in relation to stem mass (Jensen et al., 2011). The best-known PH genes are the semi-dwarfing loci *Rht-B1* and *Rht-D1* on group 4 chromosomes, conferring insensitivity to the phytohormone gibberellin (Flintham et al., 1997). Other major genes conferring reduced height are *Rht8* on 2D (Korzun et al., 1998) and the photoperiod regulator *Ppd-1* (Shaw et al., 2012). Yet several other QTLs were reported to affect PH. Griffiths et al. (2012) highlighted 16 mQTLs causing modification in PH in a meta-QTL study of four double-haploid populations. Furthermore, a recent study from Zanke et al. (2014) employing the newly available 90K iSelect wheat chip and 732 SSR markers in a genome wide association study (GWAS) of 358 European winter wheat varieties reported 280 significant markers spread over the entire wheat genome, suggesting that a wide range of loci are available to breeders for modulating PH in wheat. Like PH, GY is a complex trait regulated by a plethora of interlinked metabolic networks, including heading date, photoperiod sensitivity, number of tillers, carbohydrate remobilisation, and nutrient-use efficiency, to mention some of the traits that have a downstream effect on crop yield. Several GWA studies highlighted QTLs spread throughout the wheat genome, including the aforementioned reduced height genes (Bentley et al., 2014; Bordes et al., 2014). However, trait variation was relatively small when allele frequencies were considered.

A few studies have used GWA mapping to identify genes involved in secondary cell wall metabolism (Wang et al., 2013; Rincent et al., 2014; Ramstein et al., 2015), but not in wheat. However, promising results in terms of genetic variation for traits such as enzymatic digestibility of straw (Jensen et al., 2011) and sugar release upon pre-treatment and enzymatic hydrolysis (Lindedam et al., 2012) in collections of wheat varieties were reported. This paper presents the results of a GWA study on a collection of historical Scandinavian winter wheat varieties released onto the market over a period of more than a century, allowing a wide range of genetic variations to be explored. Traits included in the study were GY, PH and monomeric sugars released after straw enzymatic hydrolysis, relevant for second-generation bioethanol production. It was possible to detect previously reported QTLs and highlight additional genetic regions of interest for the traits investigated.

## Materials and Methods

### Plant Materials and Field Trials

A collection of 100 accessions of hexaploid winter wheat (*T. aestivum* L.) – 32 from Denmark, 66 from Sweden and two from Great Britain – released between 1900 and 2006 was grown in a field trial in 2008 in Tåstrup (55.67°N; 12.30°E), Denmark (Supplementary Table S1). The collection was intended to represent the history of more than a century of wheat breeding in the Scandinavian region. The two accessions originating from Great Britain were modern commercially available varieties currently grown in the Scandinavian area. Seeds were obtained from NordGen (Nordic Genetic Resources Centre, Alnarp, Sweden). Information regarding the accessions (e.g., pedigree, year of release, country of origin) was retrieved from the SESTO database^1^ developed by NordGen and integrated with data available from the WheatPedigree database^2^ The year of release was not available for six accessions. Except for three accessions classified as landrace, the rest of the collection consisted of commercial varieties. The experiment was conducted in two completely randomized blocks using standard agricultural practices for the area. All the 100 lines were present in the two blocks thus each line was tested with two biological replicates. PH was measured for ten plants from each plot before harvesting as the distance from the soil to the base of the spike, and the data recorded averaged. Harvesting was carried out using an experimental combine harvester that separated and collected whole plot grain and straw. GY was recorded on site and reported as wet matter tons per ha^-1^. Ligno-cellulosic biomass glucose (GLU), xylose (XYL) yields and total sugars (TS = GLU + XYL) were obtained from enzymatic hydrolysis of grain-free plant material from each plot and quantified using the NREL method, as previously described in Bekiaris et al. (2015).

### Field Trial Statistical Analysis

Initially, the lme4 package (Bates et al., 2014) based on the statistical platform R (The R Core Team, 2014) was used to develop a linear mixed model to obtain the genotypes’ best linear unbiased predictors (BLUPs) for each trait recorded. The model was expressed as Y _ij_ = μ + g_i_ + b_j_ + e_ij_, where μ represents the grand mean, *g* the random effect of the i^th^ genotype, *b* the random effect of the *j*^th^ block and *e*_ij_ the residual error ∼ N(0, σ^2^). Trait repeatability (an estimate of genetic influence on a trait similar to trait heritability) was then calculated as: $R=\sigma_{g}^{2}/\left(\sigma_{g}^{2}+\sigma_{e}^{2}\right)$, where $\sigma_{g}^{2}$ stands for total genetic variance and $\sigma_{e}^{2}$ the residual variance. Subsequently the R package mvngGrAd was employed to correct raw data for spatial field variation (Technow, 2014). The package uses the spatial position of each plot in the field, defined by row and column number, to adjust phenotypic values. When a plot is considered, the package calculates the mean phenotypic value of the surrounding plots, with the diagram for the surrounding plots as in Lado et al. (2013). The calculated value represents the growing conditions of the plot considered and is used as a covariate to adjust the observed phenotypic value. The formula describing the spatial adjustment is defined as p_i_adj_ = p_i_obs_ - b(x_i_ - $\overset{-}{x}$), where *p*_i_adj_ is the adjusted phenotypic entry, *p*_i_obs_ is the observed phenotypic data, *x*_i_ is the mean of the surrounding plots, $\overset{-}{x}$ is the mean of all *x*_i_, and *b* is the regression coefficient in the general linear model p_i_obs_ = a + bx_i_. TS was recalculated based on the adjusted values for the two sugars. A second mixed linear model following the formula Y _ij_adj_ = μ + g_i_ + e_ij_ expressed above, employing a field-corrected dataset, was used to recalculate accession BLUPs and trait R. The model giving better trait repeatability values was retained for further analyses. BLUPs were also used to calculate Pearson’s correlation between traits using the *cor.prob* function implemented in the R statistical platform.

### Genotyping and Marker Selection

Genomic DNA from 93 of the varieties employed in the field trial was extracted from two leaves of seedlings grown in controlled conditions using the hexadecyltrimethylammonium bromide (CTAB) protocol (Rogers and Bendich, 1985), with further modifications as described by Orabi et al. (2014). DNA samples were sent to Diversity Array Technology Pty Ltd (Canberra, Australia^3^) for genotyping with the wheat DArTseq^®^ platform that consists of two different sets of markers: (i) genotyping by sequencing (GBS) single nucleotide polymorphism (SNP) markers obtained by sequencing the fragments derived from genome complexity reduction and subsequent SNP calling, and (ii) presence/absence variation (PAV) DArT markers referring to whether or not a defined sequenced DNA fragment was obtained after genome enzymatic digestion. Genomic sequences of fragments from both types were also available. A detailed description of the platform used to genotype the collection can be found in literature (Kilian et al., 2012). The genotyping process did not include seven wheat accessions present in the field trial. Thus all the following analyses described here were performed on a reduced dataset including 93 lines. For details on the lines genotyped, see Supplementary Table S1. Since it was assumed that all the accessions derived from completely homozygous genotypes, SNPs showing heterozygous alleles due to the detection of multiple loci were noted as missing and then markers with >10% missing data were removed. Subsequently markers were assigned to a genetic location based on the consensus map developed by Li et al. (2015). It should be noted that the consensus map contained several recombination deserts lacking polymorphic markers. Thus chromosomes 1B, 2A, 2D, 3B, 4A, 6A, 7A, and 7B were represented by two linkage groups in the consensus map (in this study all markers on 2D mapped to only one of the groups in the consensus map). For these chromosomes, markers in the two linkage groups physically located on the same chromosome appeared unlinked, thus it was not possible to assign relative positions to the two groups. This was taken into account during further analysis. Markers were recoded as binary based on minor allele frequency (MAF) and missing genotype data were imputed using the R package scrime (Schwender, 2012), based on the five weighted nearest varieties present in the dataset. Markers with MAF below 5% were eventually removed.

### Linkage Disequilibrium Analysis, Population Structure, and GWAS

Average intra-chromosomal linkage disequilibrium (LD) decay was calculated using TASSEL v. 3.0.169 (Bradbury et al., 2007). Given the different nature of the two sets of markers, calculations were run separately for SNPs and PAVs. As reported by Li et al. (2015), the consensus map used here was created based on three crosses sharing the common parent PBW343. This variety, popular in South Asia, is known to harbor the 1B/1R translocation. According to the WheatPedigree database^4^, three varieties in the collection studied here possessed the translocation. In order to avoid any distortion in results, average interchromosomal LD decay was not calculated for chromosome 1B. Finally, chromosomes containing a recombination desert, thus constituted by two different linkage groups, were considered as separate chromosomes. Pairwise marker *r*^2^ estimates of LD were calculated and results below a significance threshold of *p* < 0.05 were discarded. *r*^2^-values were subsequently plotted against the distance in cM between the pairs of markers considered. To establish a threshold for LD decay, the 95th percentile of *r*^2^-values for unlinked markers (i.e., markers more than 50 cM apart) was calculated (Zhou et al., 2012), then a second-degree smoothed curve for the data points was fitted using the R program (The R Core Team, 2014). The projection of the interception between the fitted curve and the LD decay threshold onto the *x*-axis was assumed to determine the average chromosomal LD extent in the population considered. Chromosome 1B was analyzed separately to verify the presence of additional varieties with the rye translocation. Since the 1B consensus map was constituted by two linkage groups covering approximately 165 and 65 cM, and in consideration of long-range LD being expected as a consequence of the translocation, only markers mapped to the longest linkage group of 1B were considered. Principal components analysis (PCA) was performed on 531 PAVs, which constituted 85% of the total available genotypic information for the linkage group. Population stratification present in the collection was studied using principal coordinate analysis (PCoA) implemented in the R package ape (Popescu et al., 2012). Finally, GWAS was performed to identify positive marker-trait associations (MTA) for the five traits included in the study using the R package GAPIT (Lipka et al., 2012). BLUPs obtained from the field trial for the 93 genotyped varieties were employed as a phenotypic data to perform the association mapping study. Three principal components were employed to control for population structure and an EMMA uncompressed kinship matrix to account for cryptic relatedness. For convenience, chromosomes constituted by markers separated into two linkage groups were considered to be contiguous since this did not affect the final results. GWAS was also performed using TASSEL v. 5.2.15 (Bradbury et al., 2007) using the MLM function. This was done in order to confirm results obtain with GAPIT. Similar parameters to correct for population structure were used, i.e., three PCs as fixed effect and a kinship matrix as random effect calculated with the scaled Identity by State (IBS) method (Endelman and Jannink, 2012).

## Results

### Phenotypic Data

A summary of raw and adjusted-by-field effects on five traits recorded on the 100 historical winter wheat varieties is reported in **Table 1**. A wide range of values was observed for PH (field-adjusted data: 54–122 cm) and GY (field-adjusted data: 2.06–6.61 t ha^-1^), while conversion of ligno-cellulosic biomass into monomeric sugars showed less variation. The effect of spatial adjustment could be observed in particular for the PH and GY coefficient of variation %, which was reduced from 18.17 and 24.08 to 16.14 and 18.24, respectively. A comparison of calculated trait repeatability (R) showed how values improved when the field spatial variation correction was applied, except for GLU yield where no effect was observed. Thus, field-adjusted phenotypic data were used to calculate BLUPs and correlation coefficients. Repeatability estimates for the five traits recorded showed that PH and GY had the highest values, at 0.75 and 0.53, respectively, while traits related to biomass conversion, GLU, XYL, and TS, showed low repeatability at 0.09, 0.09, and 0.11, respectively. Pearson’s correlation (**Table 2**) between traits revealed a negative correlation between PH and GY (*r* = –0.36, *P* < 0.001) and a strong correlation between GLU and XYL (*r* = 0.57, *P* < 0.001). Moreover, sugar yield was moderately positively correlated with PH. The values observed were 0.24^∗^, 0.25^∗^, and 0.28^∗∗^ for GLU, XYL and TS, respectively. GY was also negatively correlated with GLU and TS (*r* = –0.40, *P* < 0.001 and *r* = –0.27, *P* < 0.01, respectively).

**Table 1:** Summary statistics and trait repeatability estimates for the phenotypes recorded.

		Mean		Min		Max		*SD*		CV (%)		*R*	
Trait	*n*	raw	adj	raw	adj	raw	adj	raw	adj	raw	adj	raw	adj
PH (cm)	200	96.11	96.08	52	54	127	122	17.46	15.51	18.17	16.14	0.60	0.75
GY (wm t ha–^1^)	198	4.67	4.67	1.8	2.06	8	6.61	11.25	8.52	24.08	18.24	0.40	0.53
GLU (g/g dm)	200	0.258	0.258	0.175	0.169	0.317	0.312	0.019	0.019	7.69	7.36	0.09	0.09
XYL (g/g dm)	200	0.223	0.223	0.157	0.146	0.292	0.283	0.019	0.018	8.85	8.32	0.02	0.09
TS (g/g dm)	200	0.481	0.481	0.331	0.315	0.593	0.593	0.035	0.033	7.46	7.03	0.06	0.11

*PH, plant height; GY, grain yield; GLU, glucose release upon pre-treatment and enzymatic hydrolysis; XYL, xylose release upon pre-treatment and enzymatic hydrolysis; and TS = GLU + XYL. Wm, wet matter; dm, dry matter; n, number of samples; SD, standard deviation; CV (%) coefficient of variation (SD^∗^100/mean); R, trait repeatability estimates; raw, calculated based on data not corrected for field spatial variation; adj, calculated on data adjusted for field spatial variation.*

**Table 2:** Pearson’s correlations between genotype best linear unbiased predictors (BLUPs; *n* = 100).

	PH	GY	GLU	XYL	TS
PH	1	–0.36^∗∗∗^	0.24^∗^	0.25^∗^	0.28^∗∗^
GY		1	–0.40^∗∗∗^	–0.06	–0.27^∗∗^
GLU			1	0.57^∗∗∗^	0.89^∗∗∗^
XYL				1	0.88^∗∗∗^
TS					1

*PH, plant height; GY, grain yield; GLU, glucose release upon pre-treatment and enzymatic hydrolysis; XYL, xylose release upon pre-treatment and enzymatic hydrolysis and TS, total sugars released (GLU + XYL). ^∗^p < 0.05; ^∗∗^p < 0.01; ^∗∗∗^p < 0.001.*

### Genotypic Data

Genotyping the historic Scandinavian wheat collection with the DArTseq platform resulted in an initial dataset comprising 38131 markers (12083 SNPs and 26048 PAVs). After filtering for markers with >10% missing data points, 18484 markers (4624 SNPs, 13824 PAVs) remained available. The consensus map for the DArTseq platform consisted of 28646 markers. Based on marker ID it was possible to map 6104 markers (21.3%), including 4675 PAVs and 1429 SNPs. Final filtering for MAF <5% resulted in 5525 markers used for further analysis. Their chromosomal distribution can be found in **Table 3**. The total map length covered was 3217.02 cM. The D genome was confirmed to be the least polymorphic, with only 756 markers covering 351.58 cM, while the B genome presented the highest number of markers. Within the linkage groups, 1D and 7D presented the biggest gaps between markers at 47.76 and 42.81 cM, respectively.

**Table 3:** Descriptive data of the 5525 markers used in this study.

Chr.	N. of Markers	Length covered (cM)	Biggest gap (cM)^§^
1A	270	202.57	24.14
1B^∗^	632	228.15 (164.75 + 63.4)	18.42
1D	22	78.04	47.76
2A^∗^	230	159.33 (127.39 + 31.94)	15.85
2B	536	269.48	18.39
2D	465	30.66	4.05
3A	243	250.85	27.55
3B^∗^	457	328.43 (222.01 + 106.42)	12.14
3D	96	52.25	12.14
4A^∗^	252	150.55 (41.14 + 109.41)	16.57
4B	142	94.29	13.71
4D	25	41.14	9.86
5A	126	130.83	16.06
5B	412	287.15	35.82
5D	34	20.18	13.22
6A^∗^	296	158.44 (133.66 + 24.78)	11.43
6B	386	222.71	25.83
6D	75	24.57	6.57
7A^∗^	409	190.49 (24.56 + 165.93)	27.71
7B^∗^	378	192.17 (135.25 + 56.91)	17.97
7D	39	104.74	42.81
A total	1826	1243.06	27.71
B total	2943	1622.38	35.82
D total	756	351.58	47.76
**Total**	**5525**	**3217.02**	**47.76**

*^∗^Chromosomes with two linkage groups. Genetic length of each group is indicated in parenthesis. ^§^ Within linkage group.*

### Linkage Disequilibrium and Population Structure

**FIGURE 1:**
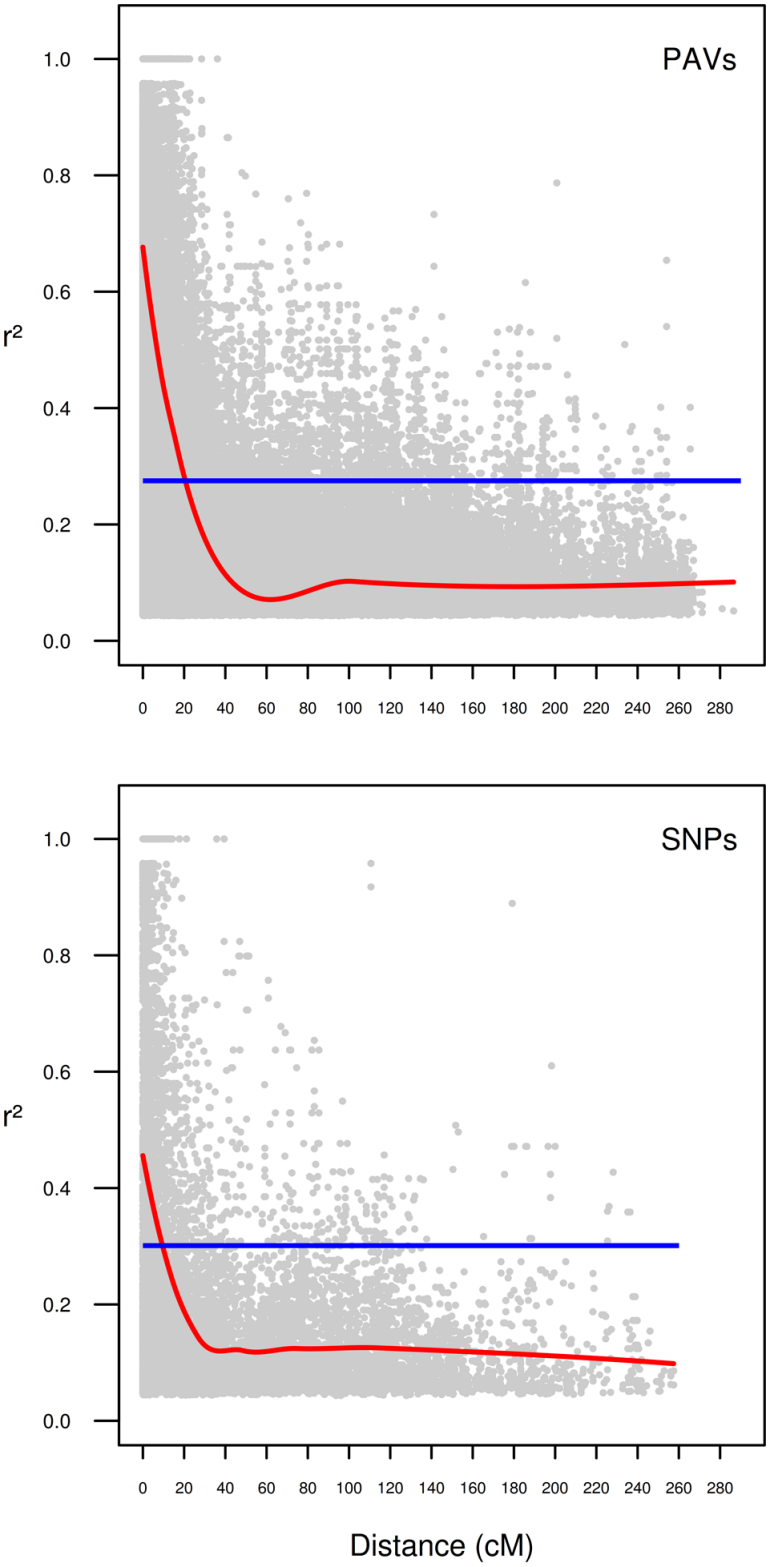
**Intra-chromosomal linkage disequilibrium (LD) decay in the historical Scandinavian winter wheat collection separated for type of marker.** Markers on chromosome 1B were omitted from calculations. *r*^2^-values of LD are plotted as a function of the distance between pairs of markers considered. Blue line: *r*^2^-values of the 95th percentile for unlinked (>50 cM) markers. Red line: second-degree smoothed loess curve.

**FIGURE 2:**
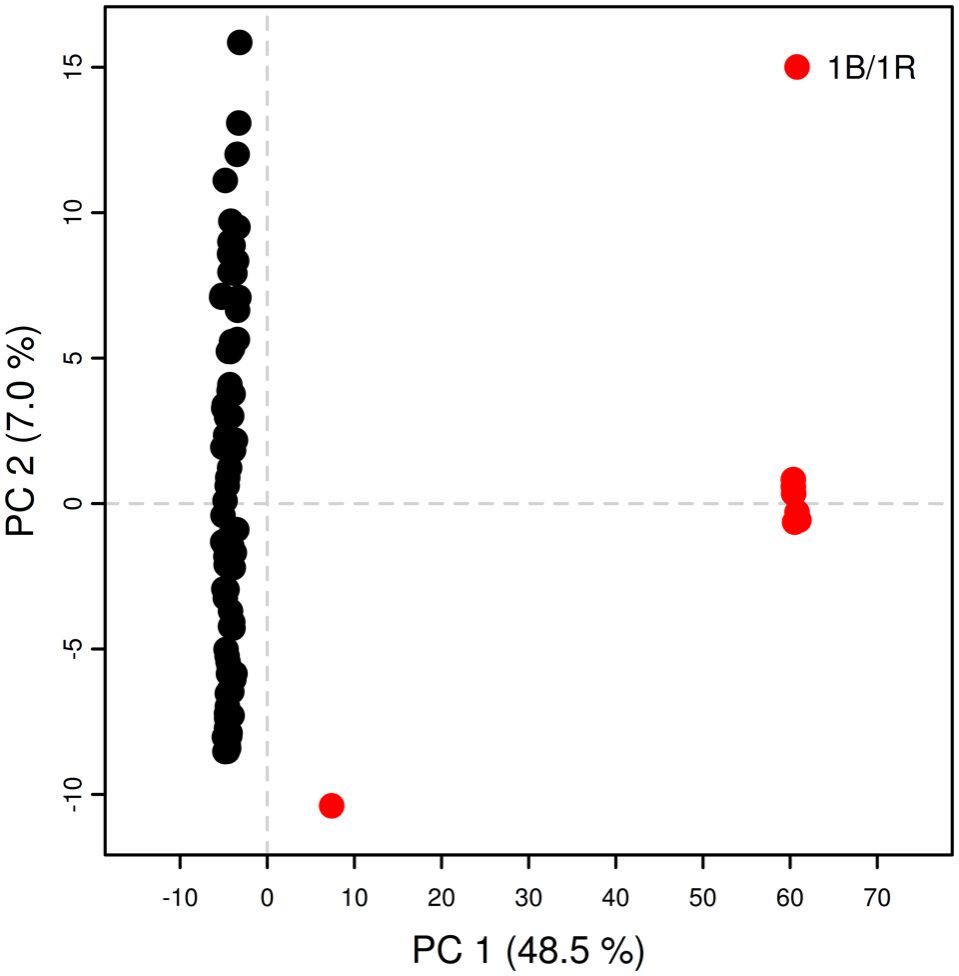
**Principal component analysis for PAVs on 1B linkage group 1.** Red circles: lines presenting 1B/1R translocation; black circles: remaining lines.

In the present collection of wheat varieties, LD was analyzed by calculating pairwise marker *r*^2^ for each chromosome. A total of 231033 *r*^2^-values (217923 for PAVs and 13110 for SNPs) were below the significance threshold (*p* < 0.05) and employed for the analysis. Of these, 56344 (∼24%) concerned markers that were considered unlinked, i.e., more than 50 cM apart. The *r*^2^ threshold for considering pairs of markers to be in LD or not was determined to be 0.27 for PAVs and 0.30 for SNPs. The second-degree smoothed loess curve calculated fell below the critical *r*^2^-value at 20.59 and 9.51 cM for PAVs and SNPs, respectively (**Figure 1**). These values were considered as the average extent of LDs for this wheat collection. PCA analysis on PAVs mapped on the 1B linkage group revealed a separation of lines according to PC1 based on the presence or absence of the 1B/1R translocation from rye (**Figure 2**). SNPs were not included given the different LD decay pattern shown between the two sets of markers. In the collection, only three varieties (“Sleipner,” “Lone,” and “Tjelvar”) were previously reported to harbor such translocation. These lines were characterized by positive values on the first PC, which represented almost half of the total variance. Based on this criterion, four additional lines showing a similar clustering pattern were detected as carrying the rye translocation. These lines were “Brandt,” “Probat,” “Galicia,” and “Kirsten” for which rye translocation had not previously been reported. Although known to carry the translocation, the “Tjelvar” variety was not located as far from the remaining genotypes on the score plot as the other six 1B/1R lines.

**FIGURE 3:**
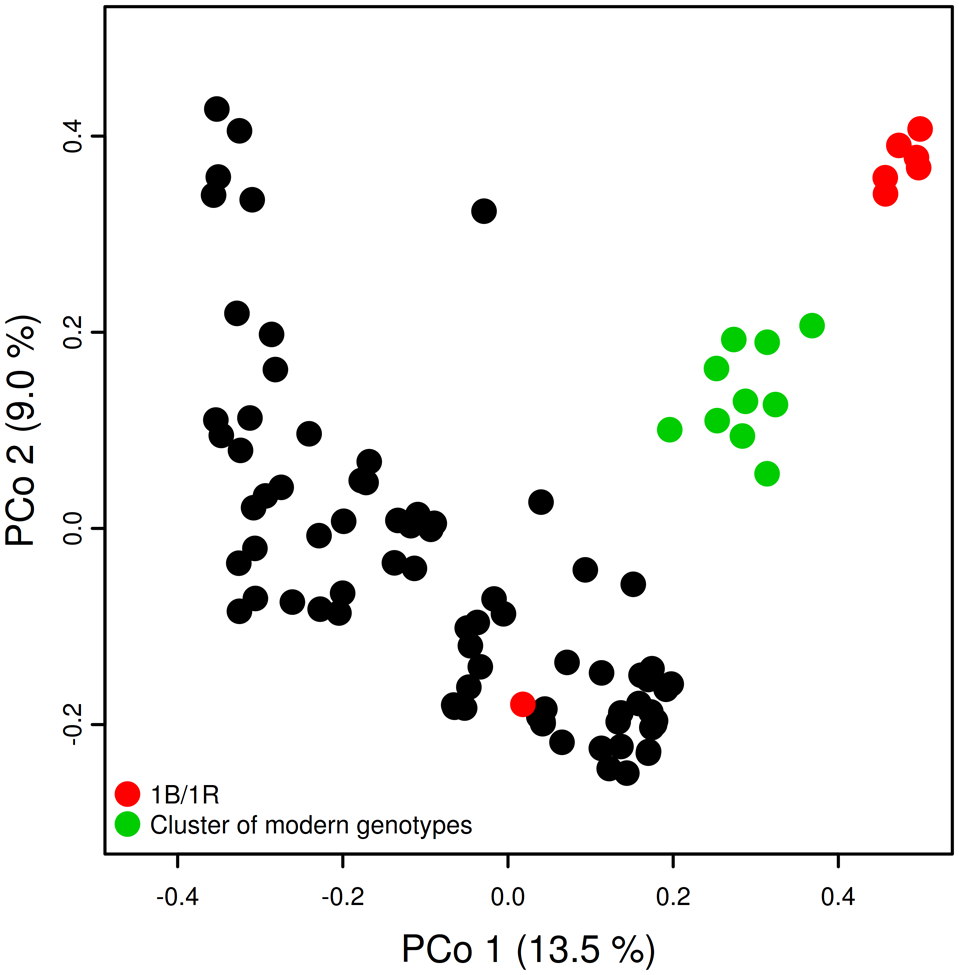
**Principal coordinate analysis of population structure using genotypic data.** Red circles: lines presenting 1B/1R translocation; green circles: cluster of modern varieties; black circles: remaining lines not showing a specific clustering pattern.

A study of the population structure using PCoA confirmed the 1B/1R translocation as a source of stratification (**Figure 3**). An analysis of the PCoA plot revealed a second group consisting of modern wheat varieties and an additional group constituted by landraces together with some old and modern varieties without any apparent differentiation according to year of release.

### Genome-wide Association Analysis for Agronomical Traits and Ligno-cellulosic Sugar Released

**FIGURE 4:**
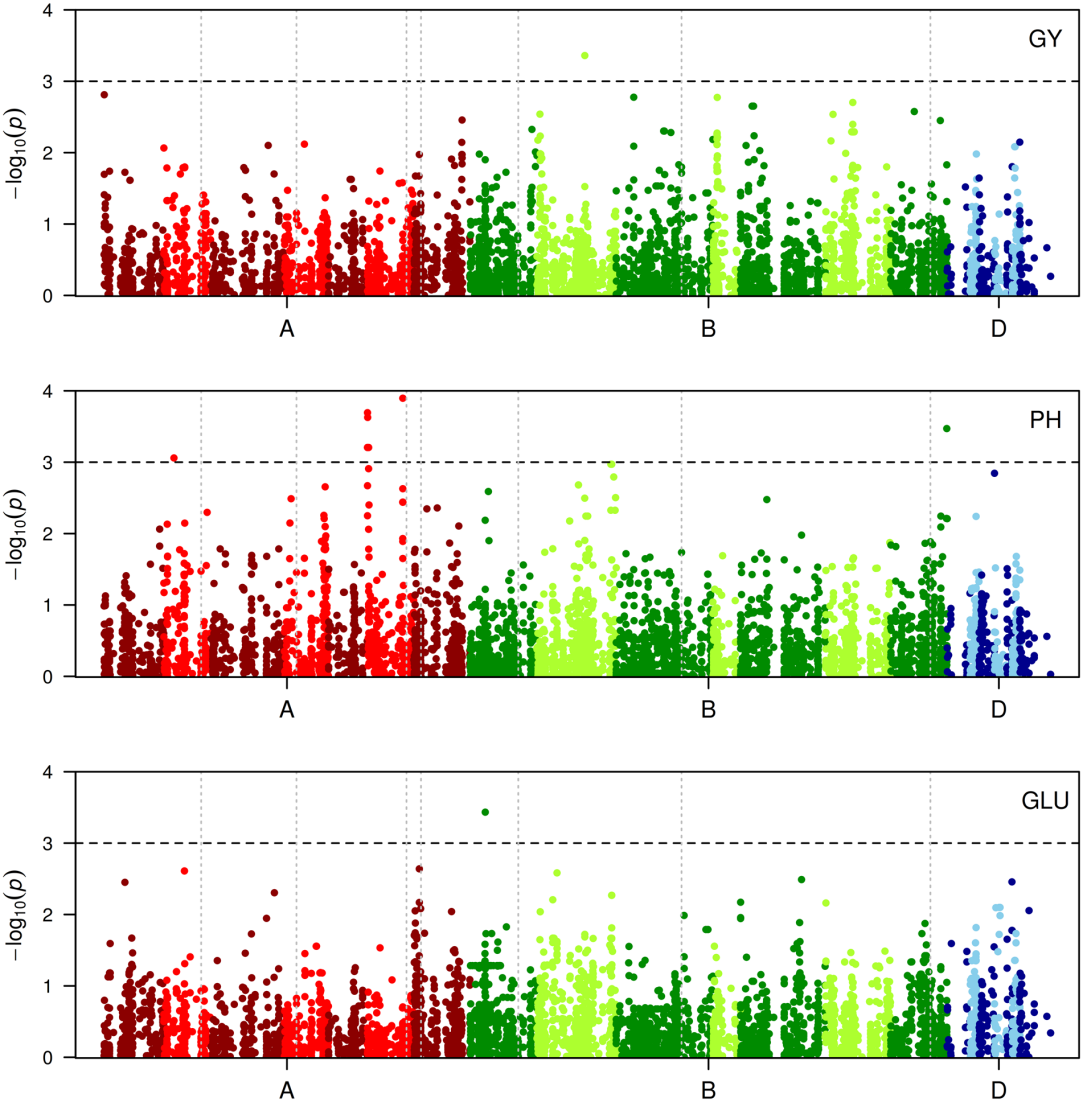
**Manhattan plots for genome wide association study (GWAS) of GY (grain yield), PH (plant height), and GLU (glucose released after enzymatic hydrolysis of biomass).** On the *x*-axis the A, B, and D genomes are in red, green, and blue, respectively. Different color tones correspond to different chromosomes within the same genome from 1 to 7. Chromosomes containing two linkage groups are represented by vertical dotted lines separating them. The dashed horizontal line indicates the significant threshold at –log_10_(*p-value*) = 3.

**FIGURE 5:**
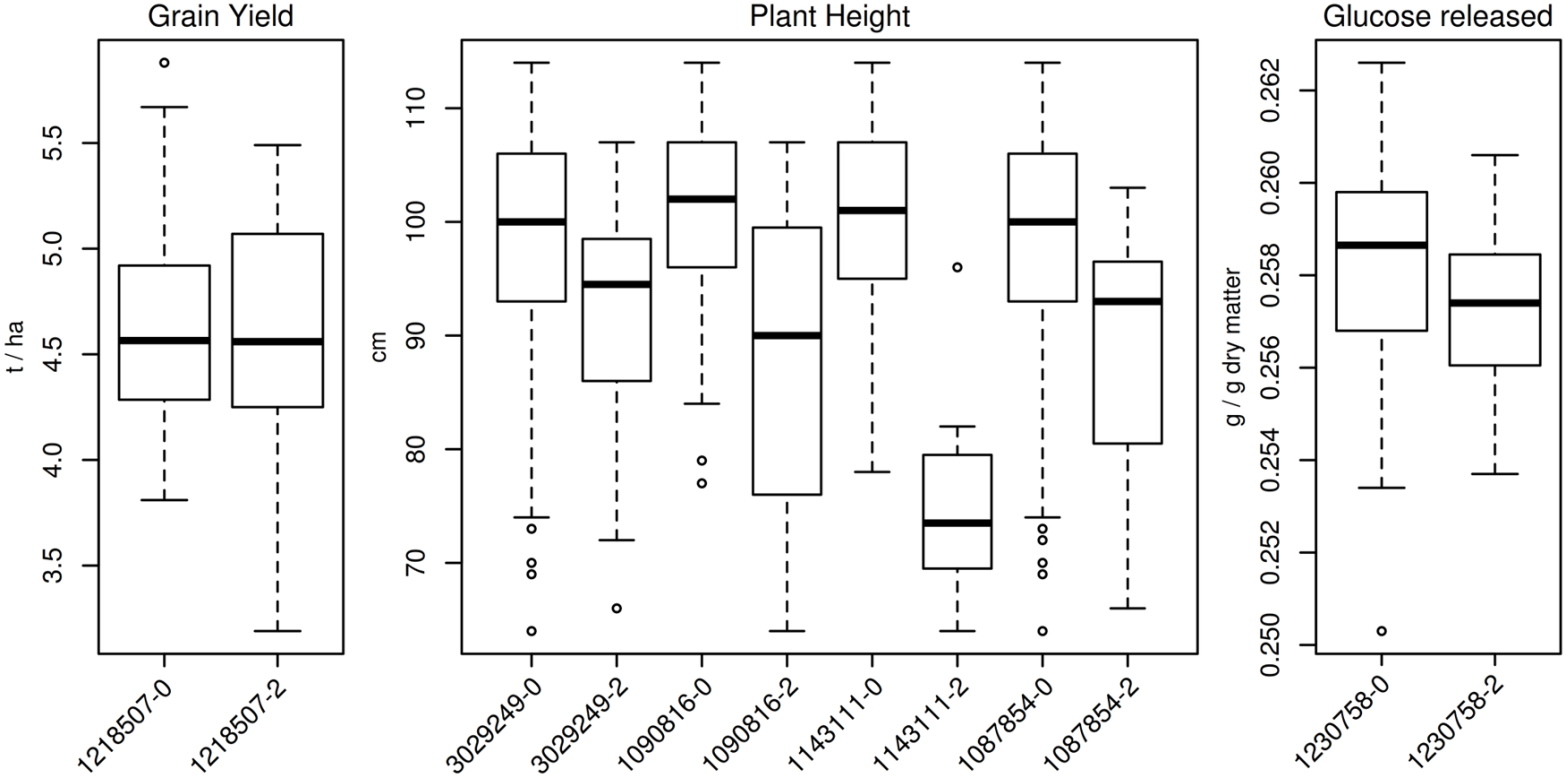
**Boxplot of trait variation for genotypes showing different alleles at significant markers.** For plant height QTL on 6A between 1.14 and 4.89 cM, only the most significant marker out of seven (SNP 1090816) is shown.

A summary of the results of the GWAS obtained using GAPIT is presented in **Table 4** and manhattan plots are given in **Figure 4**. None of the associations detected was significant when the false discovery rate (FDR) adjusted *p*-values were considered. Thus, it was decided to arbitrarily consider as significant any MTA with –log10(*p*-value) > 3 as in Houston et al. (2014). GWAS performed with TASSEL v. 5.2.15 confirmed the results from GAPIT with neglectable differences in significance p values (data not shown). No additional markers were detected as significant for the chosen –log10(*p*-value) threshold. For GY only one PAV marker was significant, located at 162.6 cM on 2B. For PH, GWAS detected ten MTAs. Of these, seven seem to detect a single QTL located on 6A between 1.14 and 4.89 cM. The remaining MTAs for PH were located on 2A (34.86 cM), 6A (121.14 cM), and 7B (linkage group 2, 55.61 cM). For sugar release, GWAS on the GLU trait showed one significant MTA at 52.52 cM on chromosome 1B (linkage group 1). The other sugar trait studied (XYL and TS) did not show any significant association with any marker. For the most significant MTA for PH on 6A at 121.14 cM, genotypes with the minor allele for PAV-1143111 were on average 25 cm shorter than genotypes with the major allele. Varieties possessing the height-reducing minor allele were mostly released onto the market in the 1990s. The remaining trait variation (PY, PH, and GLU) based on allele frequency for the QTL identified is reported in **Figure 5**.

**Table 4:** Summary results of genome wide association study (GWAS).

Trait	Marker type – Clone ID	Chr.	Pos. cM	-log_10_(*p*)	*R*^2^	MAF
GY	PAV-1218507	2B	162.60	3.36	0.12	0.27
PH	SNP-3029249	2A	34.86	3.06	0.05	0.13
	SNP-1090816	6A	1.14	3.69	0.07	0.29
	PAV-1210312	6A	1.71	3.63	0.07	0.25
	PAV-1073294	6A	1.14	3.21	0.06	0.24
	PAV-1117272	6A	1.14	3.21	0.06	0.24
	PAV-2300007	6A	1.14	3.21	0.06	0.24
	PAV-1078008	6A	4.89	3.21	0.06	0.24
	PAV-1279296	6A	4.89	3.21	0.06	0.24
	PAV-1143111	6A	121.14	3.90	0.07	0.13
	SNP-1087854	7B_2	55.61	3.47	0.06	0.08
GLU	PAV-1230758	1B_1	52.52	3.43	0.14	0.42

## Discussion

### Field Trial

From the analysis of the 100 historical winter wheat varieties, it was clear that grain production and PH were changing in response to the varieties’ year of release, while the amount of sugars obtained after pre-treatment and enzymatic hydrolysis of straw was much less affected. Indeed, spatial adjustment for variation in growth conditions improved the model’s accuracy and subsequently the repeatability estimates. Lado et al. (2013) reported similar improved heritability results using the same R-based package mvngGrAd, based on an extensive hexaploid wheat field trial study. The necessity of correction for spatial effects in field trials has been extensively discussed in a study of sorghum and for plant breeding in general (Piepho et al., 2008; Leiser et al., 2012). Other approaches have been suggested to improve output precision from field trial mixed models. Designing field experiments to control for environmental effects has always been an issue in breeding programmes for evaluating genotypes. The advantage of the approach used here is the possibility of reducing unwanted effects due to field heterogeneity after the field tests have been performed without having knowledge *a priori* of the sources of non-genetic variation.

As expected, GY and PH were the traits with the highest values of repeatability, as these traits are known to be highly influenced by genetic factors. Trait repeatability reported here for PH (0.60 and 0.75 adj, in **Table 1**) was comparable with that reported by Jia et al. (2013) for a population of wheat RIL (recombinant inbred lines) grown in four environments in China (*H* = 0.65) and the heritability estimate of 0.85 obtained by Wurschum et al. (2015) in the analysis of a collection of European winter wheat varieties. Similarly, GY repeatability (*R* = 0.53, **Table 1**) was within the range of what reported in analogous studies (Guo et al., 2015; Sukumaran et al., 2015). As expected, GY and PH were also negatively correlated as a consequence of the introduction of semi-dwarf varieties starting from 1970s and 1980s and guaranteeing higher production (Donmez et al., 2001; Shearman et al., 2005).

With regard to the traits related to second-generation bioethanol production, the results presented here showed a trend of general low repeatability estimates. In comparison, Lindedam et al. (2012) reported relatively higher heritability for wheat straw conversion of 37, 71, and 57% for C6 and C5 carbohydrates and total sugars, respectively, in a study of 20 modern wheat varieties grown in one year at two locations. In another study, Jensen et al. (2011) found a heritability of 29% for ruminant digestibility, which is also relatively high. In contrast, Larsen et al. (2012) found no significant phenotypic variation between modern cultivars in terms of sugar release upon pre-treatment and enzymatic hydrolysis. The low repeatability for sugars released reported here was, however, not completely surprising. Plant cell wall composition and bioconversion properties are known to be highly influenced by external factors as growing conditions (Gall et al., 2015). The study of the correlation between GLU and XYL released highlighted the presence of a general recalcitrance to saccharification but the low variance captured as genotypic effect would discourage the design of breeding programmes to improve carbohydrate yield for bioethanol production.

The positive correlation observed between GLU and XYL with PH as well as the negative correlation between GLU and GY led to speculation that an increase in recalcitrance occurred in modern winter wheat varieties compared to old ones. In contrast a recent study of 106 winter wheat varieties from two growing sites in Denmark, reported straw digestibility as being negatively correlated with PH (–0.36 and –0.22 for the two sites, respectively; Jensen et al., 2011). This was explained by the fact that leaves, that are more digestible, constitute a larger fraction of the straw of short-stem phenotypes. Lindedam et al. (2012) instead observed a positive correlation between PH and straw conversion as reported here and concluded that the quality of the stem, leaf and ears was more relevant for conversion than the ratio between plant anatomical parts. In those studies the varieties investigated were all relatively modern varieties. In the wheat collection analyzed in the present study a larger variation in PH was observed and therefore clear correlations between PH and sugar conversion should be expected. Additional studies, including multiple growing conditions should allow a better determination of genotype influence on cell wall related traits and shed light on the relationship between PH and plant cell wall recalcitrance.

### Genotypic Platform, LD, and Population Structure

The DArTSeq^®^ platform employed here to genotype the winter wheat collection was confirmed as an efficient tool for GWA scans and QTL mapping studies. Given the availability of a dense consensus map (Li et al., 2015), it was possible to map 5525 markers with a total map length of 3217 cM, comparable to the POPSEQ map develop by the International Wheat Genome Sequencing Consortium (Mayer et al., 2014). Despite the recent introduction of new genotypic platforms for wheat, e.g., the 90 k and the 9 k SNPs chip (Cavanagh et al., 2013; Wang et al., 2014), the DArTseq platform still maintains some advantages for genetic profiling: it is cheap, does not require prior information of the target genome and, after the recent establishment of next-generation sequencing (NGS) techniques, the platform has become an extremely high-throughput technology.

Study of LD is a prerequisite for evaluating a collection of genotypes, determining marker density needed for GWA study and defining genomic regions in the search for candidate genes related to the trait studied once marker-trait associations have been identified. Average inter-chromosomal LD decay was determined to be ∼20 cM for PAVs, which is in agreement with the report by Nielsen et al. (2014) using the previous version of the DArT platform to study a collection of European bread wheat genotypes. The values of LD decay reported here showed how the dominant PAV exhibited approximately twice the LD extent compared to the co-dominant SNP markers. This confirmed how a relatively smaller number of PAV markers are required to cover the entire wheat genome compared to SNP markers, known to exhibit more rapid LD decay, although both were equally distributed over the entire genome.

The study of population structure prior to GWA mapping revealed a moderate level of stratification in the collection, partly due to the presence of a group of lines carrying the rye translocation on chromosome 1B and partly due to a cluster of modern varieties. Similar results of moderate population stratification were reported elsewhere (Lopes et al., 2015; Naruoka et al., 2015) studying different collections of winter wheat.

### QTLs for Plant Height and Grain Yield

To identify QTLs involved in the regulation of the phenotypic traits analyzed here, a genome-wide association approach was employed. It was, however, not possible to identify significant associations when FDR correction was applied. This was probably due to the reduced number of varieties considered for the GWAS. Subsequently we choose an arbitrary significant threshold at –log10(*p*) > 3 (Gurung et al., 2014; Houston et al., 2014; Shu and Rasmussen, 2014). This resulted in the identification of a total of 12 significant MTAs involved in three out of the five traits considered. PH was the one producing the majority of positive results, with 10 MTAs on chromosomes 2A, 6A, and 7B. PH is one of the most studied phenotypes in wheat due to its involvement in plant architecture and ultimately in GY. Along with semi-dwarfing genes several other QTLs are reported to affect PH. Among the many significant markers associated with PH reported by Zanke et al. (2014) studying a collection of European winter wheat varieties, a significant MTA was detected using SSR on 6A at a similar position to that found here (∼93.5 cM). Additionally, in a recently published study, Wurschum et al. (2015) examined the genetic control of PH in a European winter collection, including genotypes from the Scandinavian area, employing the DArTseq^®^ platform also used here. A major QTL was detected on 6A (clone ID 1066954), reported to be located at 94.8 cM. In the consensus map utilized for the current study, the same clone was mapped at 121.14 and although that particular marker was not present here, a different marker (clone ID 1143111) was reported mapping at the same position, leading to the conclusion that the two major QTLs coincided. Interestingly no QTLs were highlighted on chromosomes known to harbor the dwarfing genes *Rht-D1, Rht-B1, Rht8*, and the *Ppd-D1*. This could be due to the limited number of accessions considered, which may have influenced the results from GWAS, or the lack of markers covering the genomic regions of interest, in particular for the D genome which was poorly covered. Moreover, since to some extent population structure was related to the year of release and PH was significantly correlated with the year of release, a correction for population structure during GWAS could have reduced the effect of markers in LD with major dwarfing genes, as observed in a study of maize (Larsson et al., 2013). Wurschum et al. (2015) reported the almost complete absence of the *Rht-B1* dwarfing allele for 42 varieties from Denmark, while the majority carried the short allele for *Rht-D1*. Indeed, the lack of major dwarfing genes in the collection cannot be excluded, nor their limited presence, which could have remained undetected given the parameters applied for marker filtering and the reduced number of lines included in the study.

Like PH, GY is an extremely complex trait regulated by a number of metabolic networks. Many traits have a downstream effect on crop yield. Several GWA studies highlighted QTLs spread throughout the whole genome, including the aforementioned reduced height genes (Bentley et al., 2014; Bordes et al., 2014). However, trait variation was relatively small when allele frequencies were considered. Wheat chromosome 2B is known to harbor the photoperiod insensitivity gene *Ppd-B1* (Beales et al., 2007), influencing heading time, tiller number, PH, and spikelet number, although the effects appeared less pronounced compared to *Ppd-D1* on chromosome 2D (Kamran et al., 2014). The genomic sequence of *Ppd-B1* was retrieved from the NCBI database^5^ (ID: DQ885757) and a BLAST program was run on the wheat genome assembly in the Ensembl database^6^ The best hit was located on 2B at ∼17.8 Mbp (ID: 100%; E-val:0.0). Subsequently a BLAST program was run on the DNA sequence of the significant marker identified for GY, PAV-1218507, provided by Li et al. (2015). The best hit was located on 2B, but at ∼293 Mbp (ID: 100, E-val: 4.4E-24), making the presence of strong LD between *Ppd-B1* and the significant marker identified highly unlikely. Despite the high trait repeatability observed here for GY, it is possible that the reduced number of genotypes considered hampered the possibilities of QTL detection.

### GWAS for Biomass Conversion to Bioethanol

One of the main objectives of the present study was to verify the viability of a GWA scan to identify QTLs involved in the production of second-generation biofuel. To do this, a collection of winter wheat varieties was tested in the field and the harvested ligno-cellulosic biomass phenotyped for monomeric sugars released after enzymatic hydrolysis. Repeatability for these traits was low, suggesting difficulties in identifying genetic effects given the high influence of environmental factors. In fact, a single marker trait association was reported for GLU released after biomass enzymatic hydrolysis. No MTAs were detected for XYL or total sugars released. For the GLU released, the low level of significance and low difference in trait values observed between the two allele classes necessitate further studies to confirm their validity. Furthermore, as pointed out by Oakey et al. (2013), large-scale phenotyping experiments on such traits would need advanced statistical data modeling to remove errors due to variables such as environmental factors and laboratory batch effects. In the aforementioned paper, trait heritability increased from values comparable to those reported here to up to *h*^2^= 0.50, when the optimal model was tested. A higher number of varieties as well as a multi-environment field trial could have improved the overall detected genotypic effect and subsequent GWAS results. However, this study is one of the first on plants aimed at identifying QTLs involved in biofuel production. Given the limited number of scientific publications using GWA mapping to identify genes involved in secondary cell wall metabolism (Wang et al., 2013; Rincent et al., 2014; Ramstein et al., 2015), it is clear that different approaches have so far been taken to identify such major genes. Up to now, bioinformatic techniques coupled with comparative genomics, gene silencing and plant transformation have yielded most of the knowledge about plant cell wall biosynthesis toward the production of crops with reduced biomass recalcitrance (Chen and Dixon, 2007; Sumiyoshi et al., 2013; Sundin et al., 2014). However, given the complexity of plant cell wall structures, it is necessary to study cereal crops at field scale (Alexandersson et al., 2014). Thus, field trials and genome-wide association mapping are important strategies when aiming to improve sugar yield for biofuel production. Temperate cereals such as wheat and barley are characterized by complex large genomes and draft sequences have only been released in recent years (Mayer et al., 2011, 2014). With constant advances in genotypic platforms and statistical tools for data analysis, an increased amount of knowledge regarding the complex gene networks involved in plant cell wall deposition can be anticipated.

## Conclusion

In this study, a collection of winter wheat representing more than a century of breeding efforts in the Scandinavian area was analyzed. The genetic material was tested in a single field trial, recording agronomically relevant traits such as yield and PH as well as traits related to second-generation biofuel production. The results showed reduced biomass recalcitrance to enzymatic hydrolysis of old varieties compared to new ones, and overall the possible difficulties of implementing breeding programmes aimed at improving second-generation biofuel production. However, it was possible to identify QTLs and genomic regions related to GY, PH, and GLU released from straw. In light of the fast-paced growing genomic resources available for wheat, these QTLs constitute a starting point for future investigations into the underlying causal genes responsible for trait variation.

## Conflict of Interest Statement

The authors declare that the research was conducted in the absence of any commercial or financial relationships that could be construed as a potential conflict of interest.

## Footnotes

1 http://www.nordgen.org/index.php/en/content/view/full/2

2 http://wheatpedigree.net/

3 http://www.diversityarrays.com/

4 http://wheatpedigree.net/

5 "http://www.ncbi.nlm.nih.gov/

6 http://plants.ensembl.org/Triticum_aestivum/
